# Estimating the Impact of Influenza Vaccination on Acute and ICU Hospital Bed Usage in an Influenza Season under Endemic COVID-19 in the US

**DOI:** 10.3390/vaccines10111908

**Published:** 2022-11-11

**Authors:** Van Hung Nguyen, Joaquin F. Mould-Quevedo

**Affiliations:** 1 VHN Consulting Inc., Montreal, QC H2V3L8, Canada; 2 Seqirus USA Inc., Summit, NJ 07901, USA

**Keywords:** influenza vaccine, COVID-19, hospital bed occupancy, ICU occupancy, cell-based influenza vaccine, adjuvanted influenza vaccine

## Abstract

In 2021–2022, influenza vaccine coverage in the US dropped below pre-COVID-19 pandemic levels. Cocirculation of COVID-19 and influenza could place a substantial burden on hospital utilization in future seasons, particularly given the reduced exposure to influenza during the pandemic. We used a dynamic susceptible-exposed-infected-recovered model to simulate influenza transmission with varying influenza vaccine coverage against a background of COVID-19 circulation, in order to estimate acute and ICU hospital bed occupancy for both diseases. We evaluated two vaccine scenarios: egg-based quadrivalent influenza vaccine (QIVe) for all age groups or cell-based QIV (QIVc) for 0.5–64 year-olds with adjuvanted QIV (aQIV) for ≥65 year-olds. ICU bed availability was more limiting than general hospital bed availability, with a vaccine coverage of ≥70% required to avoid negatively impacting ICU bed availability in a high-incidence influenza season. The timing of disease peaks was a key factor together with vaccine coverage, with a difference of ≥50 days needed between peak influenza and COVID-19 bed usage together with 65% influenza vaccine coverage to avoid negative impacts. QIVc + aQIV resulted in lower bed occupancy which, while not substantial, may be critical in very high hospital resource usage situations. In a situation with co-circulating influenza and COVID-19, proactive vaccination planning could help to avert overwhelming healthcare systems in upcoming influenza seasons.

## 1. Introduction

Vaccination remains a critical health tool to both reduce the morbidity and mortality caused by influenza and to minimize the impact on healthcare services. In the 2020–2021 season, influenza vaccine coverage rates in the US reached an all-time high of 52.1% of all individuals aged 6 months or older, and 50.2% of adults aged ≥ 18 years [1]. However, during the 2021–2022 season, vaccination rates dropped to below pre-COVID-19 pandemic levels, with only 45.4% of adults vaccinated [2,3]. Reduced coverage compared with previous years was particularly notable among ethnic minorities and individuals aged ≥ 65 years, who are considered at higher risk of influenza complications [4]. A number of factors could have contributed to this fall in vaccine uptake including directly overlapping COVID-19 primary series and booster vaccination campaigns, which in part overshadowed the influenza vaccination campaign; apathy following a season of unusually low influenza circulation; reluctance for COVID-19 and influenza vaccine co-administration; and a general uninterest in receiving any additional vaccinations (vaccine fatigue).

Seasonal influenza vaccines vary in their effectiveness from season to season, based partly on the degree of match with circulating strains and timing of virus circulation relative to vaccination [5,6,7]. Choice of the optimal vaccine for each age group, together with increasing coverage rates, particularly in high-risk groups, could substantially reduce the pressure on healthcare services, increasing ward and intensive care unit (ICU) bed availability for other patients and reducing healthcare worker absence [8,9]. Enhanced vaccines, i.e., adjuvanted or higher dose vaccines, have demonstrated increased efficacy compared with standard influenza vaccines in adults aged ≥ 65 years, who often experience lower vaccine efficacy owing to age-related immunosenescence [10,11,12]. Cell-based vaccine development methods can remove the potential for egg-adaption, leading to increased likelihood of a match with circulating A/H3N2 strains [13], and therefore may be particularly beneficial in situations where healthcare services are at risk of being overwhelmed during an influenza epidemic.

COVID-19 mitigation measures, including social distancing, lockdowns, and reductions in population mixing contributed to the low incidence of influenza during the COVID-19 pandemic [14]. However, relaxation of these measures, together with lowered population-level natural immunity due to reduced exposure and vaccination uptake during the 2021–2022 season could lead to a resurgence in seasonal influenza in the upcoming season. In line with this hypothesis, the Southern hemisphere saw an early and sharp increase in notifications of laboratory-confirmed influenza cases compared with recent seasons [15]. Given the similarities in trends in influenza vaccination rates in recent years [16], it is likely that the Northern hemisphere influenza season will follow this pattern, with the potential for significant strain on healthcare services if peak influenza incidence coincides with a wave of COVID-19 infections. The reduced population-level immunity means that a larger than normal proportion of the population will be vulnerable to symptomatic influenza infection, likely resulting in more hospitalizations than are seen in a standard influenza season. In addition, there is much uncertainty about the evolution and epidemiology of SARS-CoV-2, including the effectiveness of vaccines against future circulating variants, and the proportion of the population vulnerable to severe disease and hospitalization. However, it is likely that in upcoming seasons, both COVID-19 and influenza will substantially burden healthcare services, with vaccination remaining a key tool to reduce severity of both diseases. In this analysis, we simulate the effect of varying influenza vaccine coverage for two different vaccine scenarios on the hospital resource utilization amidst a background of COVID-19 circulation.

## 2. Methods

### 2.1. Model Structure

We simulated influenza transmission using an age-structured, four-strain (A/H1N1, A/H3N2, B/Victoria, and B/Yamagata) susceptible-exposed- infected-recovered (SEIR) dynamic model, which has been used previously for influenza epidemiology modelling in the US (see Supplementary Materials for further details) [17]. A dynamic model was chosen as it was assumed that vaccination has an impact on disease transmission. In line with previous analysis, we assumed 27% of the population started the season (pre-vaccination) protected from influenza infection (subclinical or clinical), and neither infection- nor vaccine-induced protection waned during the season [18]. Vaccination was modelled by removing individuals from the susceptible (S) to the recovered (R) component of the model (Figure S1), with vaccine effectiveness assumptions varying by age group and vaccine type (Table S1). For the contact matrix, we used the mean daily time of exposure between age groups in the US, estimated from Prem et al. [19]. Data from two influenza seasons (2011–2012 and 2017–2018) in the US [20], reflecting inter-seasonal variation in influenza incidence (low and high incidence, respectively), were used to calibrate the model and estimate the number of symptomatic infections per day (Table S2). In total, 60% of infections were assumed to be symptomatic [21]. A fraction of symptomatic infections required hospitalization, with the probability of hospitalization assumed to be the same as used in a previous analysis (ranging from 0.0006 to 0.0421, dependent on age group) [22]. We assumed that 10% of hospitalized patients would require intensive care across age groups [23,24].

Total US acute (i.e., non-ICU) hospital bed and ICU bed capacities were estimated at 1,000,000 and 100,000 based on published estimates (Table 1) [25,26]. A baseline occupancy rate of 70% was assumed [27], thus leaving 300,000 hospital and 30,000 ICU beds open for influenza and COVID-19 patients. Durations of influenza-related hospital and ICU stay were assumed to be 5 and 7 days, respectively [23,28]. The base case COVID-19 epidemiological scenario utilized the mean of peak hospital and ICU occupancy values across three variants (Alpha, Delta, and Omicron), based on US epidemiological data [29]. Differences between variants using daily hospitalizations and ICU stays from Alpha, Delta, and Omicron separately were considered in the sensitivity analysis (see below).

### 2.2. Influenza Vaccine Scenarios

Two different influenza vaccine scenarios were evaluated in the base case analysis. The first scenario (scenario 1) assumed that an egg-based quadrivalent influenza vaccine (QIVe) was administered, irrespective of age. In the second scenario (scenario 2), recipients aged 6 months to 64 years received a cell-based QIV (QIVc) and recipients ≥ 65 years received an adjuvanted QIV (aQIV). The second scenario was chosen based on the estimated reductions in hospitalizations from use of QIVc versus QIVe observed in previous modelling of US data, and estimates of the effectiveness of QIVc (in 6 month- to 64 year-olds) and aQIV (in ≥65 year-olds) [17,30]. Parameters for vaccine effectiveness of QIVe were based on US data from 2011 to 2020, giving a mean estimate of 42% across age groups, strains, and years [2]. Mean vaccine coverage rates were estimated for a coverage of the population aged ≥ 6 months of between 40% and 70%, and weighted by age group and population size (Table S3) [1]. For the base case analysis, a mean coverage rate of 45% was used, based on the observed coverage from the 2021–2022 season [1].

Scenario analyses were conducted using multiple vaccine coverages (40–70%), vaccine effectiveness estimates and COVID hospitalization dynamics across variants. In scenario 2 we assumed the relative effectiveness of QIVc versus QIVe to be 10% [31], and aQIV vs. QIVe to be 25%, based on extrapolated data from adjuvanted trivalent influenza vaccines (aTIV) [30,32]. Furthermore, we assumed that the influenza and COVID-19 circulation occurred simultaneously, with peaks in bed occupancy occurring on the same day.

### 2.3. Sensitivity Analysis

Sensitivity analysis was performed to evaluate the impact of the time of peak bed occupancy of COVID-19 (50 and 5 days before influenza peak bed occupancy) and the use of data from individual COVID-19 variants (Alpha, Delta, and Omicron) in place of average data as models for COVID-19 bed usage. In addition, we varied the probability of hospitalization rates in acute and intensive care, duration of hospital stay, and vaccine effectiveness within the ICU from half to 1.5 times base case values.

### 2.4. Model Development

The dynamic transmission model was developed in R 4.2.1 and C++ with a Shiny package interface. It used mainly the following packages and corresponding libraries: Rcpp 1.0.9, RcppArmadillo 0.11.2.3.1, and RcppGSL 0.3.11. In addition, it relied on the package nloptr for model calibration.

## 3. Results

**Figure 1:**
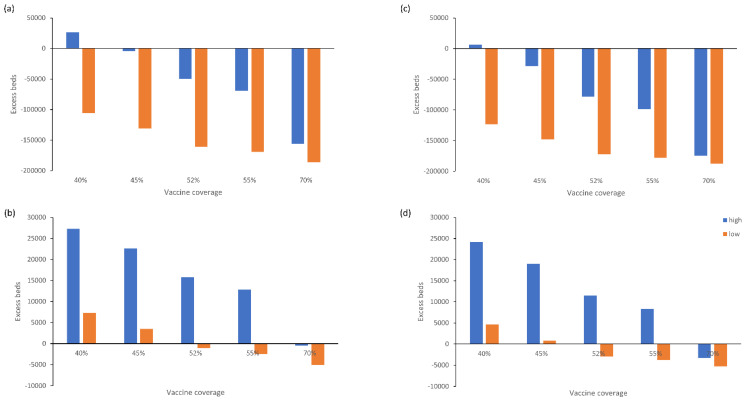
Excess (**a**) acute and (**b**) ICU beds used for treatment of COVID-19 and influenza patients by vaccine coverage (base case scenario: QIVe for all age groups) and (**c**) acute and (**d**) ICU beds used for treatment of COVID-19 and influenza patients by vaccine coverage (scenario 2: QIVc for 6 months to 64 years, aQIV for ≥65 years) during high and low incidence influenza seasons.

Based on the influenza vaccine coverage observed in the 2021–2022 season (45%) and base case COVID-19 bed occupancy estimates averaged across the three variants, scenario 1 (in which all recipients received QIVe) resulted in a peak hospital bed occupancy by COVID-19 and influenza patients of 295,379 and an ICU occupancy of 52,608 in a high influenza incidence season, or 169,122 and 33,477, respectively, in a low incidence season (Figure 1a,b; Table S4). Therefore, in both these situations, ICU bed capacity (30,000) would be exceeded.

While the number of acute hospital beds would not be limiting in a low incidence season, at least 45% influenza vaccine coverage would be needed in a high incidence season to avoid exceeding acute bed capacity. In contrast, ICU bed availability was limiting in most scenarios, with a vaccine coverage of 70% or more needed to avoid exceeding bed capacity in a high incidence season, and 52% or more in a low incidence season.

The findings were similar for the second vaccination scenario (QIVc + aQIV) across both high and low incidence seasons, however, the absolute total number of beds occupied by COVID-19 and influenza patients was lower compared with scenario 1 for the same vaccine coverage rates (Figure 1c,d, Table S5). As with the base case scenario, a vaccine coverage of roughly 70% or more would be needed to avoid exceeding ICU bed capacity in a high incidence season, and 52% or more in a low incidence season.

**Figure 2:**
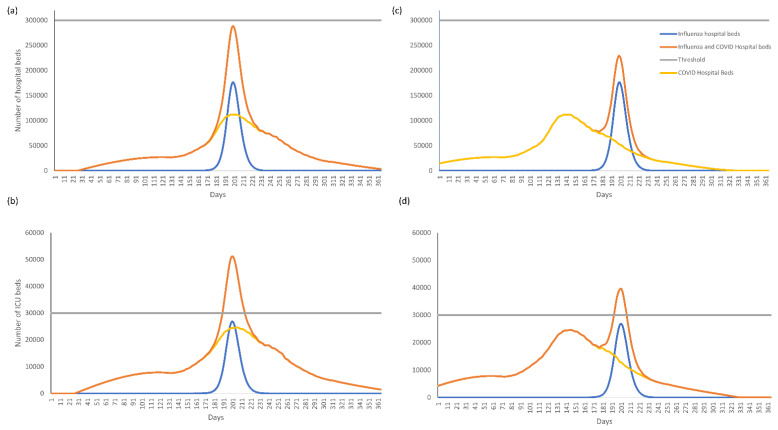
(**a**) Acute and (**b**) ICU bed occupancy over time for the base case scenario (QIVe for all age groups) where peak COVID-19 and influenza bed occupancy coincide and (**c**) acute and (**d**) ICU bed occupancy where peak COVID-19 bed occupancy occurs 50 days before peak influenza bed occupancy. Assumes an influenza vaccine coverage of 45% and high incidence influenza season.

**Figure 3:**
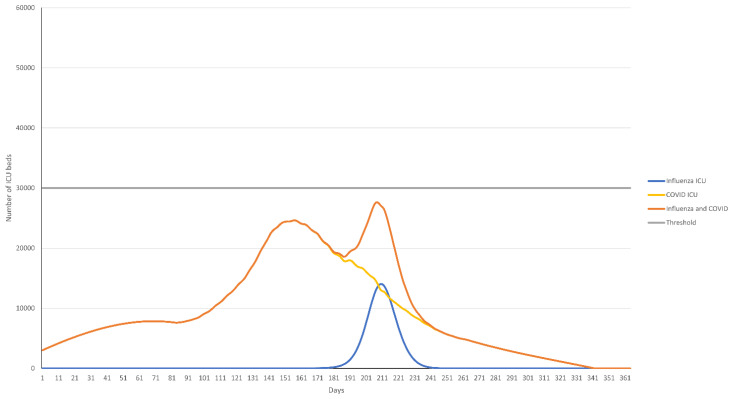
ICU bed occupancy over time for the base case scenario (QIVe for all age groups) where peak COVID-19 bed occupancy occurs 50 days before peak influenza bed occupancy and assuming an influenza vaccine coverage of 65%.

The timing of the peak bed occupancy of COVID-19 and influenza also substantially influenced the total number of beds occupied. In a high influenza incidence season where the peaks of COVID-19 and influenza epidemics coincide and 45% of the population has received influenza vaccination, ICU bed occupancy remained considerably over the 30,000-bed threshold for the duration of the influenza epidemic peak, whereas acute hospital beds occupied remained below the 300,000 threshold (Figure 2a,b). If the COVID-19 peak bed occupancy occurred 50 days or more before the influenza peak, total ICU bed occupancy was reduced but remained above 30,000 (Figure 2c,d). A vaccination rate of 65% was need to reduce ICU bed occupancy below the 30,000 threshold (Figure 3).

Sensitivity analysis based on individual COVID-19 variant waves showed the same trends as with the average wave across variants, with the exception that acute bed utilization was also exceeded with an Omicron-like wave (Figures S2 and S3). Irrespective of the influenza incidence, a reduced probability of hospitalization, shorter length of stay, and improved vaccine effectiveness reduced the peak occupancy of COVID-19 and influenza total acute and ICU beds (Table 2), with similar findings across vaccine scenarios.

## 4. Discussion

The results of our analysis indicate that ICU bed availability is a major limitation when influenza and COVID-19 are co-circulating. Assuming typical historical COVID-19 hospital resource usage during a variant wave, an influenza vaccine coverage of 70% or more would be necessary to avoid exceeding ICU bed availability during a high-incidence influenza epidemic. With vaccine coverage rates of ~40–45%, there would likely be insufficient acute hospital beds in this scenario too. However, these estimates come with the caveat that they are based on the total of number of beds available, irrespective of the availability of healthcare workers. Through the first two years of the COVID-19 pandemic, sick or quarantined healthcare workers also limited availability of hospital care [33], so it is possible that higher vaccine coverage rates would be needed in a combined COVID-19-influenza season than those estimated in this analysis. They are also of course based on assumptions that future COVID-19 and influenza epidemics may be similar to past epidemics of each pathogen.

While the use of QIVc and aQIV instead of QIVe across all age groups did not have a substantial impact, it did provide some leeway in bed availability which may be critical in stopping healthcare systems being overwhelmed. In this analysis, acute and ICU bed usage was dominated by COVID-19 patients, with relatively low available capacity for patients with influenza. Therefore, while absolute numbers of beds made available by the QIVc + aQIV vaccine scenario were not large, the proportion of the total influenza beds saved was substantial, particularly at higher coverage rates. This may be particularly important in a season dominated by A/H3N2, with many cases having been recently reported in the Southern hemisphere [34,35,36]. A/H3N2 causes the greatest burden of disease in older adults who are already at high risk of complications [37], leading to high rates of hospital and ICU bed occupancy. Additionally, the potential for egg-adaptation of A/H3N2 vaccine virus strains could reduce vaccine efficacy [38,39], thereby also impacting hospital bed usage.

Our analysis indicates that the timing of the peak incidence of hospitalization from influenza and COVID-19 is more important than the precise hospitalization dynamics of individual COVID-19 variants. While the variants in this analysis differed in their impact on hospital bed usage, sensitivity analysis indicated that effect on peak bed occupancy was moderate compared with the timing of peak bed occupancy. In a situation where peak bed usage differs by at least 50 days between COVID-19 and influenza, the model suggests that while there would be a substantial reduction in bed occupancy, this gap would still not be enough to avoid exceeding ICU bed usage based on a vaccine coverage rate of 45%. In this situation, a coverage rate of approximately 50% would be needed to reduce ICU bed occupancy below the 30,000 threshold. However, as mentioned previously, other factors such as availability of healthcare workers and organization of care would likely also play a role, further limiting acute and ICU bed availability, meaning that in reality a larger gap may be needed to avoid negatively impacting healthcare.

As with all simulations, this analysis had a number of limitations. Many suggest our conclusions may be optimistic for some populations. First, for simplicity, we used a fixed value to estimate total available acute and ICU beds while in reality the numbers of beds vary from year to year and are uncertain. Additionally, influenza vaccine effectiveness varies annually, and as we cannot accurately predict the effectiveness for an individual upcoming season, we relied on estimates from previous seasons. We also did not dynamically adjust vaccination rates throughout the season, and based on the model on an assumed fixed vaccine coverage throughout the influenza season. The modelling approach we used assumed the influenza and COVID-19 epidemics spanned the entire US, whereas circulation of COVID-19 and influenza varies regionally, and local COVID-19 waves were often more compressed than reflected in the national data and our variant curves. We expect this model is thus optimistic and fails to reflect the potential acuteness of local surges. Additionally, healthcare resources and utilization vary regionally, with some areas having far fewer acute hospital beds or higher occupancy than other regions [40]. While a regional analysis may have resulted in different results, particularly in regions with high numbers of hospital beds and healthcare resources, it is likely that many regions (particularly those with limited resources or high population density) would have the same or worse impacts as those seen in our base case scenario, and would likely need to turn away patients before the national capacity was met. Such heterogeneities could be amplified by correlated COVID-19 and influenza vaccination coverages. However, it is also possible that peaks of influenza and COVID-19 could vary regionally across the US, allowing the potential for co-operation and transfer of patients among regions to reduce pressure in overwhelmed regions. One further limitation is that we did not consider the association between mortality and access to care. Several studies have suggested that there is a strong correlation between limited access to care and excess mortality [41,42,43,44], therefore in a worst case scenario of co-circulation of COVID-19 and influenza, it is likely that there will also be an excess mortality rate associated with limitations in ICU facilities. In addition, we did not consider either the impact of influenza vaccination against potential co-infections or on COVID-19 disease. Finally, data were only available up to the initial Omicron wave; analysis of BA.4 and BA.5 subvariants may have led to different estimates of bed occupancy. However, given the limited impact of COVID-19 variant on overall excess bed usage, it is likely that high rates of influenza vaccine coverage would still be needed to prevent bed occupancy exceeding availability with either of these subvariants.

## 5. Conclusions

In summary, high rates of influenza vaccine coverage can reduce negative impacts on acute and ICU bed availability. Vaccination with cell-based and adjuvanted vaccines could lead to more bed availability than vaccination with standard dose egg-based vaccines. In a case where COVID-19 and influenza are co-circulating, the timing of epidemic peaks would be the major driver of maximum resource utilization. Therefore, proactive vaccination planning against both diseases, including potential coadministration in vulnerable groups, could help to avert overwhelming healthcare systems in the upcoming influenza seasons.

## Figures and Tables

**Table 1:** Model parameters.

Item	Values	References
Total available hospital beds	1,000,000	[26]
Baseline occupancy rate for hospital beds	70%	[27]
Available hospital beds for COVID-19/influenza	300,000	
Total ICU beds	100,000	[25]
Baseline occupancy rate for ICU beds	70%	[27]
Available ICU beds for COVID-19/influenza	30,000	
Duration of influenza hospitalization	5 days	[28]
Duration of influenza ICU stay	7 days	[23]
Influenza vaccine coverage	40–70%	
Vaccine effectiveness QIVe ≥ 6 months ^a^	35–58%	Table S1
Vaccine effectiveness QIVc 6 months to <65 years ^a^	44–64%	Table S1
Vaccine effectiveness aQIV ≥ 65 years ^a^	44%	Table S1

^a^ See Table S1 for details of vaccine effectiveness estimates by age group and vaccine type.

**Table 2:** Sensitivity analysis evaluating the effects of reducing the probability of hospitalization, length of stay, and vaccine efficacy. Each parameter was varied to be 50% and 150% of base case assumptions. The acute hospital and ICU bed occupancy for high and low incidence influenza seasons are shown. Analysis assumes use of QIVe across all age groups and a vaccine coverage rate of 45%.

Variable	Influenza Incidence	50% Base Case	150% Base Case
**Hospital beds**			
Probability of hospitalization	High incidence	202,094	383,079
	Low incidence	133,767	178,098
Length of stay	High incidence	185,647	390,664
	Low incidence	129,525	181,247
Vaccine efficacy	High incidence	389,546	203,375
	Low incidence	226,871	123,081
**ICU beds**			
Probability of hospitalization	High incidence	38,378	65,879
	Low incidence	28,035	34,852
Length of stay	High incidence	40,880	62,112
	Low incidence	28,587	34,137
Vaccine efficacy	High incidence	66,552	38,676
	Low incidence	42,262	26,398

## Data Availability

The data used in this study are not publicly available.

## Supplementary Materials

The following supporting information can be downloaded at: https://www.mdpi.com/article/10.3390/vaccines10111908/s1, Figure S1: Transmission model and outcome module; Figure S2: Hospital bed occupancy over time for the base case scenario (QIVe for all age groups) where peak COVID-19 and influenza bed occupancy coincide; Figure S3: ICU bed occupancy over time for the base case scenario (QIVe for all age groups) where peak COVID-19 and influenza bed occupancy coincide; Table S1: Vaccine effectiveness assumptions by age group and vaccine type; Table S2: Calibration parameters for the dynamic model of influenza transmission; Table S3: Percentage of individuals assumed vaccinated within each age group cohort in the base case scenario (overall vaccine coverage: 45%); Table S4: Influenza vaccine coverage rate (QIVe for all age groups) on ICU and hospital bed usage in a high and low incidence influenza season; Table S5: Influenza vaccine coverage rate (QIVc for 6 months to 64 years, aQIV for ≥65 years) on ICU and hospital bed usage in a high and low incidence influenza season [45,46,47,48,49,50].
