# Supplementary material for: Estimating the Impact of Influenza Vaccination on Acute and ICU Hospital Bed Usage in an Influenza Season under Endemic COVID-19 in the US
Source: Vaccines (Basel). 2022 Nov 11;10(11):1908. doi: 10.3390/vaccines10111908 (PMC9693626; doi:10.3390/vaccines10111908)
Supplement: Supplementary file 1 [file vaccines-10-01908-s001.zip › vaccines-1992566-supplementary.pdf]

Supplementary Materials

Mathematical Equations for the Dynamic Model

Differential Equations

$S^N_{t+1,i} = S^N_{t,i} - \lambda_{t,i} S^N_{t,i}$<br>$E^{1N}_{t+1,i} = E^{1N}_{t,i} + \lambda_{t,i} S^N_{t,i} - \gamma_1 E^{1N}_{t,i}$<br>$E^{2N}_{t+1,i} = E^{2N}_{t,i} + \gamma_1(E^{1N}_{t,i} - E^{2N}_{t,i})$<br>$I^{1N}_{t+1,i} = I^{1N}_{t,i} + \gamma_1 E^{2N}_{t,i} - \gamma_2 I^{1N}_{t,i}$<br>$I^{2N}_{t+1,i} = I^{2N}_{t,i} + \gamma_2(I^{1N}_{t,i} - I^{2N}_{t,i})$<br>$R^N_{t+1,i} = R^N_{t,i} + \gamma_2 I^{2N}_{t,i}$	$S^V_{t+1,i} = S^V_{t,i} - \lambda_{t,i} S^V_{tmi}$<br>$E^{1V}_{t+1,i} = E^{1V}_{t,i} + \lambda_{t,i} S^V_{t,i} - \gamma_1 E^{1V}_{t,i}$<br>$E^{2V}_{t+1,i} = E^{2V}_{t,i} + \gamma_1(E^{1V}_{t,i} - E^{2V}_{t,i})$<br>$I^{1V}_{t+i,} = I^{1V}_{t,i} + \gamma_1 E^{2V}_{t,i} - \gamma_2 I^{1V}_{t,i}$<br>$I^{2V}_{t+1,i} = I^{2V}_{t,i} + \gamma_2(I^{1V}_{t,i} - I^{2V}_{t,i})$<br>$R^V_{t+1,i} = R^V_{t,i} + \gamma_2 I^{2V}_{t,i}$
For t=0,<br>$S^N_{0,i}$ = Initial number of susceptibles<br>$I^{1N}_{0,i}$ = Initial number of infectious individuals<br>$E^{1N}_{0,i} = E^{2N}_{0,i} = I^{2N}_{0,i} = R^N_{0,i} = 0$	For t=0,<br>$S^V_{0,i} = E^{1V}_{0,i} = E^{2V}_{0,i} = I^{1V}_{0,i} = I^{2V}_{0,i} = R^V_{0,i} = 0$

where

S^N and S^V represent the number of susceptibles of age group i history. The susceptibles indexed by N are naive and the ones indexed by V have received the vaccine.

E^{1X}_i and E^{2X}_i represent exposed, but not yet infectious individuals with vaccine history X.

I^{1X}_i and I^{2X}_i represent infectious individuals with vaccine history X.

R^X_i represent immune individuals of age class I with vaccine history X

α_i is the proportion of the vaccinees become protected (we assume full protection), while the rest $1 - \alpha_i$ remain fully susceptible. The vaccine efficacy α_i depends on age group.

γ_1, γ_2 represent the rate of loss of latency (γ_1) and infectiousness (γ_2) in both groups. Hence, the average latent period is $2/\gamma_1$, and the average infectious period is $2/\gamma_2$

Force of Infection

$$\lambda^{*}_{t,1} = q\sigma i \sum_{j=0}^{100} \sum_{x=\{N,V\}} Cij \left(\frac{I^{1X}_{t,j} + I^{2X}_{t,j}}{Nj} \right)$$

$\lambda^{*}_{t,i}$ is the age-group specific force of infection

q is the transmissibility parameter,

σ_i is the susceptibility of age group i

c_{ij} is the rate at which individuals in age group i make contact with those in age group j

I^{1X}_{i} and I^{2X}_{i} represent infectious individuals with vaccine history X.

N_j is the total number of people in age group j

Figure S1. Transmission model and outcome module

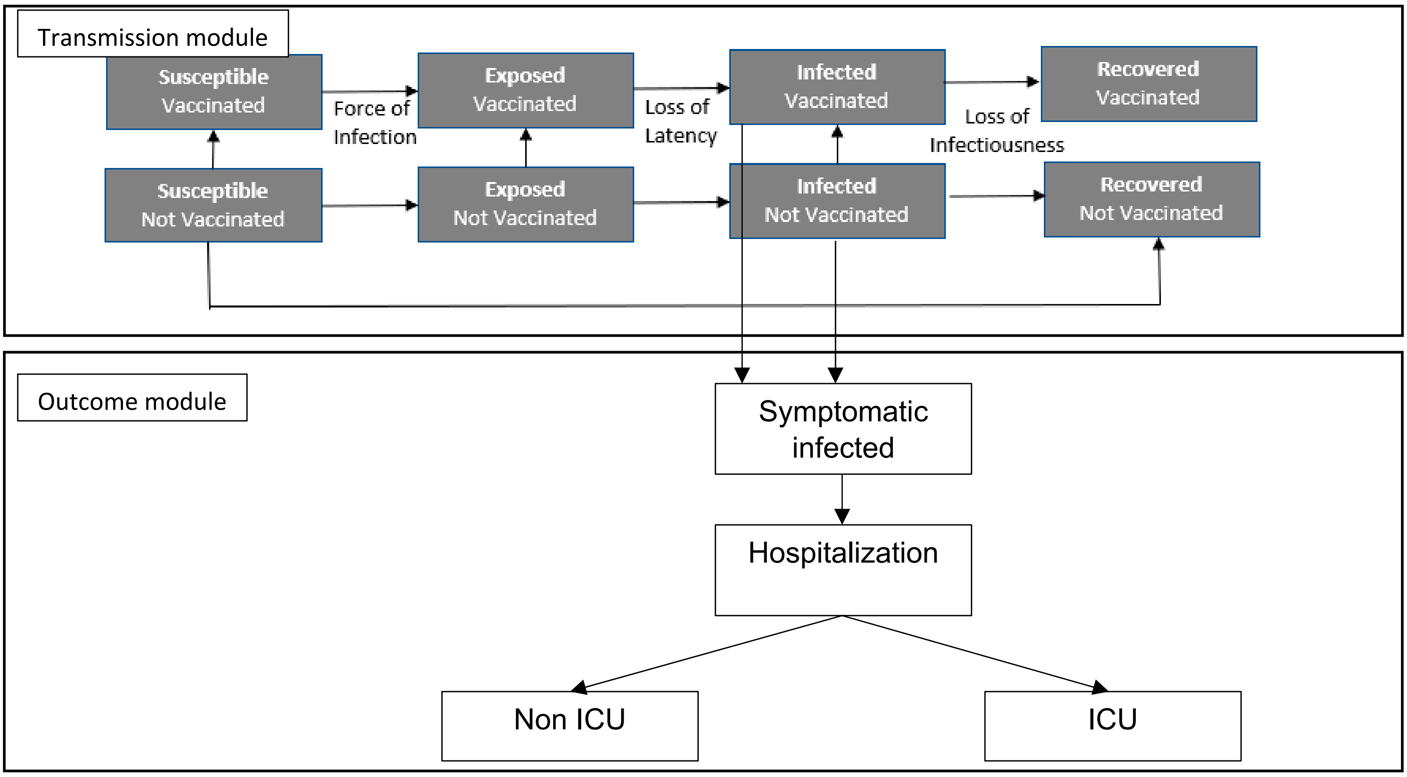

Figure S2. Hospital bed occupancy over time for the base case scenario (QIVe for all age groups) where peak COVID-19 and influenza bed occupancy coincide. Data are based on (a) alpha, (b), delta, and (c) omicron COVID-19 variants

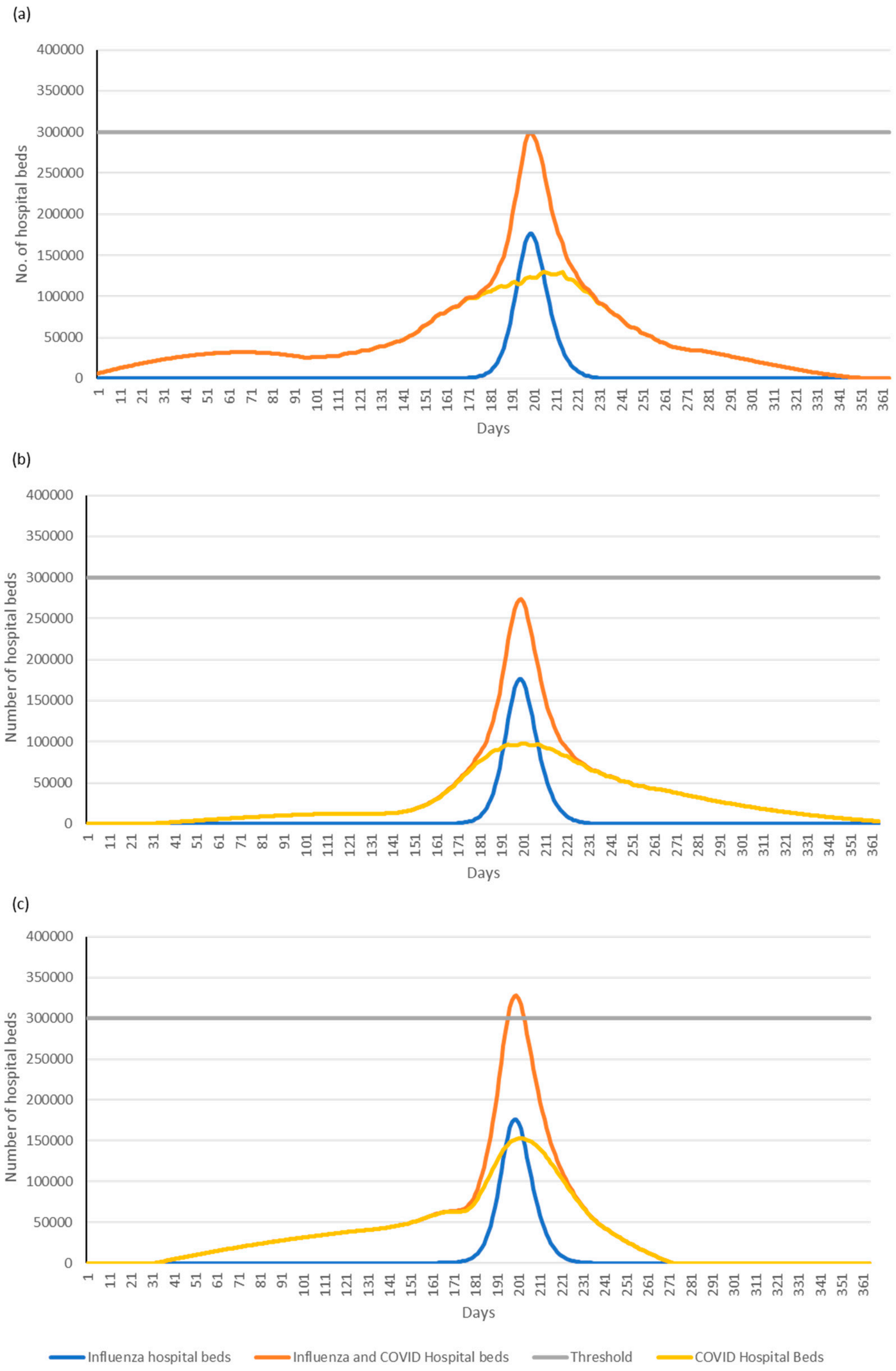

Figure S3. ICU bed occupancy over time for the base case scenario (QIVe for all age groups) where peak COVID-19 and influenza bed occupancy coincide. Data are based on (a) alpha, (b), delta, and (c) omicron COVID-19 variants

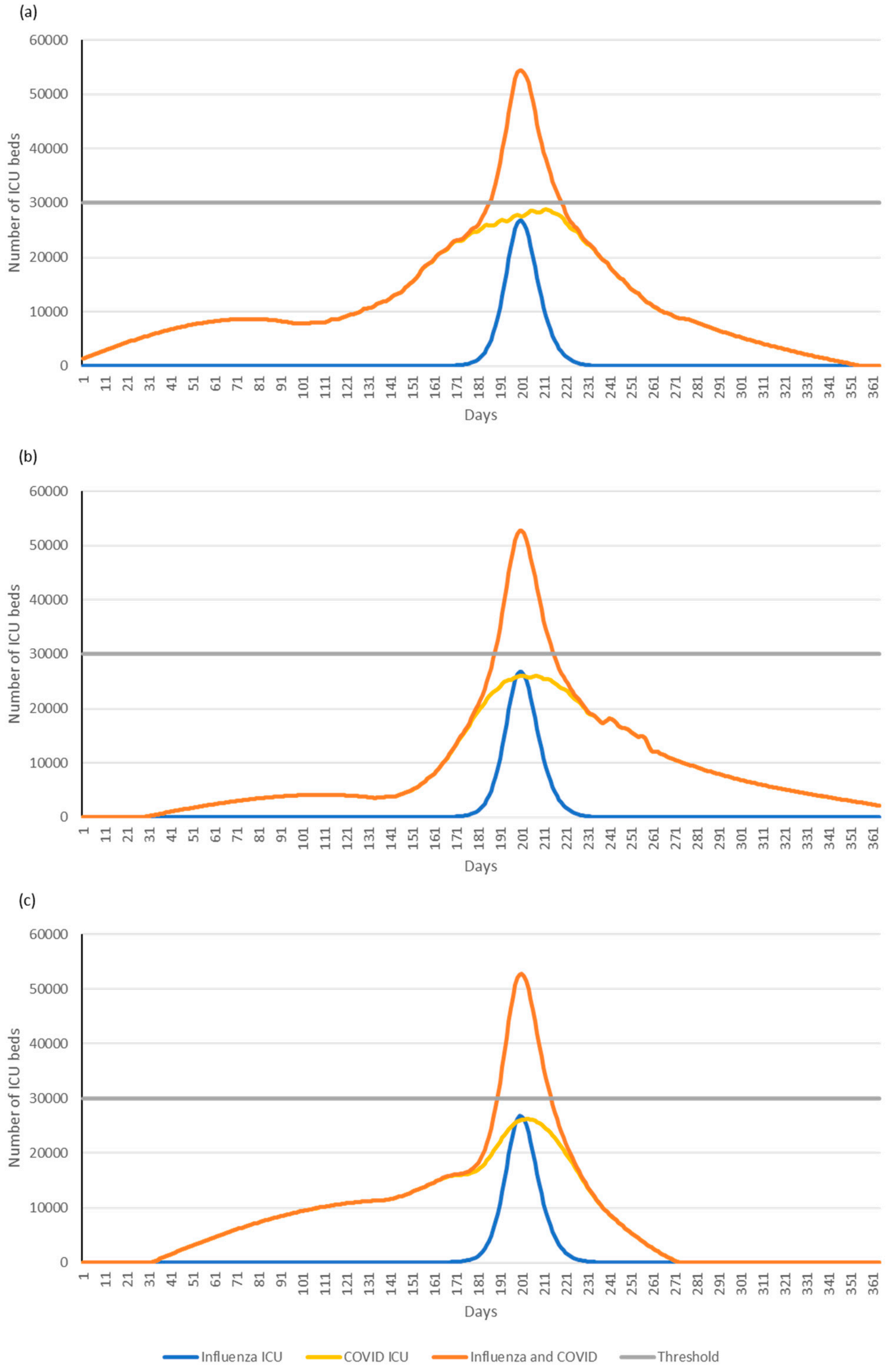

Table S1. Vaccine effectiveness assumptions by age group and vaccine type

Age group	QIVe [1]	QIVc [2]	aQIV [3]
**6months to 4 years**	42%	46%	–
**5 to 17 years**	42%	46%	–
**18 to 49 years**	42%	46%	–
**50 to 64 years**	42%	46%	–
**65 to 74 years**	42%	42%	53%
**≥75 years**	42%	42%	53%

QIVc, cell-based quadrivalent influenza vaccine; QIVe, egg-based quadrivalent influenza vaccine; aQIV, adjuvanted quadrivalent influenza vaccine

Table S2. Calibration parameters for the dynamic model of influenza transmission

Parameter	Low incidence	High incidence
R_0	2.46	3.04
$R_{eff}(t=0)$	1.80	2.22
Pre-acquired immunity*	27%	27%
Latency period, days†	0.8	0.8
Infectious period, days†	1.8	1.8
Probability of transmission upon contact		
6 months–4 years	0.114	0.188
5–17 years	0.078	0.102
18–49 years	0.102	0.105
50–64 years	0.099	0.190
65–74 years	0.104	0.107
≥75 years	0.065	0.133
Probability of hospitalization by age		
6 months–4 years	0.0141	
5–17 years	0.0006	
18–49 years	0.0042	
50–64 years	0.0193	
≥65 years	0.0421	
Probability of ICU hospitalization from influenza hospitalisation¶	0.1%	

* Pre-acquired immunity was set to be consistent with estimates from Baguelin et al. 2013 [4]

†Estimate based on Ferguson et al. 2005 [5]

¶Estimate based on Beumer et al., 2018 and Lina et al., 2020 [6,7]

Transmission parameters were estimated for each age-group using the Bound Optimization BY Quadratic Approximation (BOBYQA) [8] algorithm for likelihood maximization as implemented NLopt [9]. The number of observed influenza cases by age-group was assumed to follow a Poisson distribution. R_0 and R_{eff} were computed via the next generation matrix method [10]. Compared to R_0, R_{eff} simply accounted for background immunity at the start of the epidemic.

Table S3. Percentage of individuals assumed vaccinated within each age group cohort in the base case scenario (overall vaccine coverage: 45%)

Age group	Vaccine coverage
6 months–4 years	68%
5–17 years	59%
18–49 years	38%
50–64 years	54%
65–74 years	75%
≥75 years	75%

Table S4. Influenza vaccine coverage rate (QIVe for all age groups) on ICU and hospital bed usage in a high and low incidence influenza season. COVID-19 values based on the mean peak occupancy values across the three variants assessed

	Coverage rate	Hospital Visits	Hospital beds used Influenza	ICU beds used Influenza	Hospital beds used Influenza + COVID-19	ICU beds used Influenza + COVID-19	Excess hospital beds used Influenza + COVID-19	Excess ICU beds used Influenza + COVID-19
High incidence	40%	868 021	215 104	32 659	326 705	57 286	26 705	27 286
	45%	781 861	183 778	27 981	295 379	52 608	-4 621	22 608
	52%	642 626	138 701	21 198	250 302	45 825	-49 698	15 825
	55%	574 884	119 177	18 244	230 778	42 871	-69 222	12 871
	70%	191 566	32 110	4 947	143 711	29 574	-156 289	-426
Low incidence	40%	431 130	82 500	12 665	194 101	37 292	-105 899	7 292
	45%	324 415	57 521	8 850	169 122	33 477	-130 878	3 477
	52%	167 263	27 694	4 268	139 295	28 895	-160 705	-1 105
	55%	118 457	19 016	2 932	130 617	27 559	-169 383	-2 441
	70%	13 622	1 915	296	113 516	24 923	-186 484	-5 077

Table S5. Influenza vaccine coverage rate (QIVc for 6 months to 64 years, aQIV for ≥65 years) on ICU and hospital bed usage in a high and low incidence influenza season. COVID-19 values based on the mean peak occupancy values across the three variants assessed

	Coverage rate	Hospital Visits	Hospital beds used Influenza	ICU beds used Influenza	Hospital beds used Influenza + COVID-19	ICU beds used Influenza + COVID-19	Excess hospital beds used Influenza + COVID-19	Excess ICU beds used Influenza + COVID-19
High incidence	40%	815 709	194 534	29 595	306 135	54 222	6 135	24 222
	45%	713 614	159 768	24 380	271 369	49 007	-28 631	19 007
	52%	545 040	110 261	16 894	221 862	41 521	-78 138	11 521
	55%	463 055	89 385	13 718	200 986	38 345	-99 014	8 345
	70%	87 891	13 736	2 119	125 337	26 746	-174 663	-3 254
Low incidence	40%	355 669	65 090	10 006	176 691	34 633	-123 309	4 633
	45%	237 828	40 192	6 191	151 793	30 818	-148 207	818
	52%	101 811	16 082	2 481	127 683	27 108	-172 317	-2 892
	55%	66 246	10 157	1 567	121 758	26 194	-178 242	-3 806
	70%	6 128	804	124	112 405	24 751	-187 595	-5 249

References

[1] Centers for Disease Control and Prevention (CDC). US Flu VE data for 2019-2020. https://www.cdc.gov/flu/vaccines-work/2019-2020.html. Accessed 30 June, 2022.

[2] Boikos C, Fischer L, O'Brien D, Vasey J, Sylvester GC, Mansi JA. Relative Effectiveness of the Cell-derived Inactivated Quadrivalent Influenza Vaccine Versus Egg-derived Inactivated Quadrivalent Influenza Vaccines in Preventing Influenza-related Medical Encounters During the 2018-2019 Influenza Season in the United States. *Clin Infect Dis*. Aug 2 2021;73(3):e692-e698.

[3] Mannino S, Villa M, Apolone G, et al. Effectiveness of adjuvanted influenza vaccination in elderly subjects in northern Italy. Am J Epidemiol. Sep 15 2012;176(6):527-33.

[4] Baguelin M, Flasche S, Camacho A, Demiris N, Miller E, Edmunds WJ, Assessing Optimal Target Populations for Influenza Vaccination Programmes: An Evidence Synthesis and Modelling Study. PLoS Med 2013;10, e1001527.

[5] Ferguson NM, Cummings DA, Cauchemez S, Fraser C, Riley S, et al. Strategies for containing an emerging influenza pandemic in Southeast Asia. Nature 2005;437: 209–214.

[6] Beumer MC, Koch RM, van Beuningen D, et al. Influenza virus and factors that are associated with ICU admission, pulmonary co-infections and ICU mortality. J Crit Care. 2019;50:59-65.

[7] Lina B, Georges A, Burtseva E, et al. Complicated hospitalization due to influenza: results from the Global Hospital Influenza Network for the 2017-2018 season. BMC Infect Dis. 2020;20(1):465

[8] Powell, M.J.D. 2009. The BOBYQA algorithm for bound constrained optimization without derivatives, Department of Applied Mathematics and Theoretical Physics, Cambridge England, technical reportNA2009/06.

[9] Johnson, SG. The NLopt nonlinear-optimization package, https://nlopt.readthedocs.io/en/latest/

[10] Diekmann O, Heesterbeek JAP, Roberts MG. The construction of next-generation matrices for compartmental epidemic models. J. R. Soc. Interface. 2010;7, 873–885.
